# Data Analysis Protocol for the Development and Evaluation of Population Pharmacokinetic Models for Incorporation Into the Web-Accessible Population Pharmacokinetic Service - Hemophilia (WAPPS-Hemo)

**DOI:** 10.2196/resprot.6559

**Published:** 2016-12-07

**Authors:** Alanna McEneny-King, Gary Foster, Alfonso Iorio, Andrea N Edginton

**Affiliations:** ^1^ School of Pharmacy University of Waterloo Waterloo, ON Canada; ^2^ Department of Clinical Epidemiology and Biostatistics McMaster University Hamilton, ON Canada; ^3^ Biostatistics Unit The Research Institute St Joseph’s Healthcare Hamilton, ON Canada; ^4^ Health Information Research Unit Department of Clinical Epidemiology and Biostatistics McMaster University Hamilton, ON Canada; ^5^ Hamilton Niagara Hemophilia Program Department of Medicine McMaster University Hamilton, ON Canada

**Keywords:** hemophilia, population pharmacokinetics, factor VIII, factor IX, tailored prophylaxis

## Abstract

**Background:**

Hemophilia is an inherited bleeding disorder caused by a deficiency in a specific clotting factor. This results in spontaneous bleeding episodes and eventual arthropathy. The mainstay of hemophilia treatment is prophylactic replacement of the missing factor, but an optimal regimen remains to be determined. Rather, individualized prophylaxis has been suggested to improve both patient safety and resource utilization. However, uptake of this approach has been hampered by the demanding sampling schedules and complex calculations required to obtain individual estimates of pharmacokinetic (PK) parameters. The use of population pharmacokinetics (PopPK) can alleviate this burden by reducing the number of plasma samples required for accurate estimation, but few tools incorporating this approach are readily available to clinicians.

**Objective:**

The Web-accessible Population Pharmacokinetic Service - Hemophilia (WAPPS-Hemo) project aims to bridge this gap by providing a Web-accessible service for the reliable estimation of individual PK parameters from only a few patient samples. This service is predicated on the development of validated brand-specific PopPK models.

**Methods:**

We describe the data analysis plan for the development and evaluation of each PopPK model to be incorporated into the WAPPS-Hemo platform. The data sources and structure of the dataset are discussed first, followed by the procedures for handling both data below limit of quantification (BLQ) and absence of such BLQ data. Next, we outline the strategies for building the appropriate structural and covariate models, including the possible need for a process algorithm when PK behavior varies between subjects or significant covariates are not provided. Prior to use in a prospective manner, the models will undergo extensive evaluation using a variety of techniques such as diagnostic plots, bootstrap analysis and cross-validation. Finally, we describe the incorporation of a validated PopPK model into the Bayesian post hoc model to produce individualized estimates of PK parameters.

**Results:**

Dense PK data has been collected for more than 20 brands of factor concentrate from both industry-sponsored and investigator-driven studies. The model development process is underway for the majority of molecules, with refinement and validation to be completed in 2017. Further, the WAPPS-Hemo co-investigator network has contributed more than 300 PK assessments for use in model development and evaluation. This constitutes the largest repository of this type of PK data globally.

**Conclusions:**

The WAPPS-Hemo service aims to eliminate barriers to the uptake of individualized PK-tailored hemophilia treatment. By incorporating this tool into routine practice, clinicians can implement a personalized dosing strategy without performing rigorous sampling or complex calculations. This service is centred on validated models developed according to the robust approach to PopPK modeling described herein.

**ClinicalTrial:**

ClinicalTrials.gov NCT02061072; https://clinicaltrials.gov/ct2/show/NCT02061072 (Archived by WebCite at http://www.webcitation.org/6mRIXJh55)

## Introduction

### Background

Hemophilia is an inherited bleeding disorder caused by a deficiency in clotting factor VIII (FVIII, hemophilia A) or factor IX (FIX, hemophilia B). FVIII and FIX are key constituents in the coagulation cascade, which produces fibrin clots in response to blood vessel injury [1]. Consequently, hemophiliacs suffer from spontaneous, often recurring, joint bleeds, eventually leading to arthropathy. Hemophilia A is the more common form of the disease, affecting approximately 1 in 5000 males, while hemophilia B is considerably more rare (approximately 1 in 20,000) [2].

Modern hemophilia treatment consists of replacement of the deficient factor [3]. Replacement therapy began with the introduction of plasma-derived clotting factor concentrates in the 1960s, and advances in DNA technologies in the 1990s propelled the development of recombinant coagulation factors and the more recent design of longer-lasting recombinant products [4,5]. Clotting factor replacement therapy may be administered according to two main treatment strategies: episodic and prophylactic. The concept of prophylaxis, initiated by Nilsson and colleagues in the 1970s [6,7], is derived from the clinical observation that patients with moderate hemophilia (ie, those with clotting factor activity greater than 1% of normal) are less prone to the spontaneous bleeds and consequent arthropathy seen in those with severe hemophilia [8]. Today, there is global unanimity that prophylaxis should be initiated in young children before joint disease is apparent [9-11], as episodic treatment has been shown to be ineffective for the prevention of arthropathy [10]. However, implementation of the prophylactic approach varies considerably between countries [12]. The cost and availability of factor concentrates are major barriers to its widespread adoption, as is the challenge of patient compliance [13].

Despite its proven clinical benefit, an optimal dosing strategy for prophylaxis has yet to be determined. Evidence suggests that treatment should be individualized for best results, both from a therapeutic and economic perspective [14]. Typically, the pharmacokinetic (PK) properties of factor concentrates are assessed with classical PK studies, which are carried out in a small homogeneous group of participants, usually young and healthy. Subsequently, patients are empirically dosed by weight based on average PK estimates without taking into account individual variation in PK parameters beyond what can be predicted by age and weight [15]. Indeed, participants who appear similar may exhibit different PK behavior due to unpredictable variability. For example, Collins et al examined the variability in time to reach a critical factor level and found significant variation not only between children and adults, but within each group as well [16]. Unfortunately, performing an individual PK study with a classical approach requires 11 samples – 4 in the distribution phase (0 to 1 h) and 7 in the elimination phase (up to 48 h for FVIII, 72 h for FIX) – as outlined in recommendations from the International Society on Thrombosis and Haemostasis [17], making individualized PK-tailored dosing a difficult approach to apply in a clinical setting, especially when it involves pediatric patients.

One opportunity to overcome some of the limitations and barriers discussed above is offered by population pharmacokinetic (PopPK) modeling. Indeed, PopPK studies can make use of both rich and sparse sampling, which allows for a larger and more heterogeneous group of participants (eg, pediatric, elderly, and critical care patients) to be included due to less demanding sampling schedules [18]. Moreover, the PopPK approach allows for the partitioning of the total variability in PK response in a population into predictable and unpredictable variability. Predictable variability can be attributed to covariates that influence PK, such as body weight, age, and disease phenotype, and the identification of meaningful covariates can help to recognize at-risk subpopulations [19]. Unpredictable variability may occur both between subjects (BSV) and within a single subject (WSV), and a main goal of PopPK is to estimate the magnitude of these unexplained sources of variability so that a suitable dosing strategy may be determined [20,21]. In the case of hemophilia, WSV is small relative to BSV [15], so an individualized dosing regimen is appropriate. This approach is used in the therapeutic monitoring of several other conditions [22-24], and a 2010 study by Björkman et al indicated that a PopPK model combined with a limited sampling strategy could be as useful for the prediction of individual FVIII PK as a classical study [25]. However, adoption of this method has been hampered due to the complexity of the models needed to describe clotting factor PK and a relative shortage of PK data due to the rarity of the disease.

**Figure 1:**
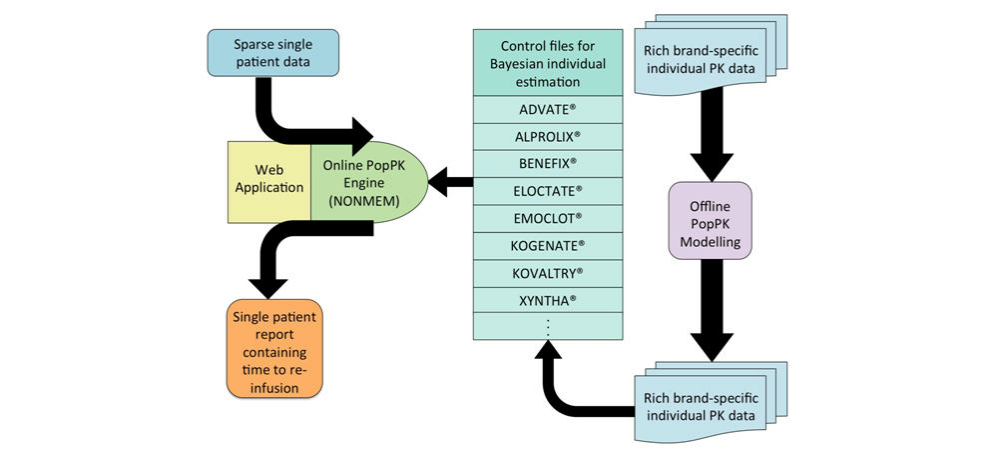
The Web-Accessible Population Pharmacokinetic Service - Hemophilia (WAPPS-Hemo) platform uses brand-specific population pharmacokinetic models and submitted patient data to generate reports of individual pharmacokinetic profiles and estimates.

In response, the Web-Accessible Population Pharmacokinetic Service - Hemophilia (WAPPS-Hemo, NCT02061072) project was launched in April 2013 at McMaster University, Hamilton, Ontario, Canada. A detailed description of the project methodology, objectives and progress is published separately [26]. In brief, WAPPS-Hemo aims at supporting clinicians in assessing individual PK for more informed dosing decisions (Textbox 1). The goal of the WAPPS-Hemo project is to set up a centralized, dedicated, Web-accessible, actively moderated service, allowing for (1) the input of anonymized and certified patient data by clinicians; (2) automatic estimation of patient-specific PK parameters; (3) expert validation of the estimation process; and (4) reporting of estimates to clinicians (Figure 1). WAPPS-Hemo represents the first non-industry sponsored, Web-based PopPK Bayesian calculator providing individualized PK estimates.

This report outlines the methods used for the development of brand-specific PopPK models, which form the knowledge base of the WAPPS-Hemo Bayesian individual forecast platform.

**The Web-Accessible Population Pharmacokinetic Service - Hemophilia project summary.**

In the framework of the population pharmacokinetic (PopPK) approach, estimating reliable outcomes by the Web-Accessible Population Pharmacokinetic Service - Hemophilia (WAPPS-Hemo) service requires that underlying PopPK models are well developed using a sufficiently large population of individuals. The WAPPS-Hemo project has assembled a vast database of clotting factors VIII and IX pharmacokinetic (PK) data across numerous brands and this represents the largest repository of this type of data globally. The repository includes PK data from industry sponsors and independent investigators; furthermore, clinical sites contributing data to the WAPPS-Hemo repository for individual estimation agreed upon subsequent use of those data for modeling. Indeed, onboarding of clinical sites requires that each participating site enters the network by signing a data transfer agreement where the site commits to data provision and takes responsibility for clinical use of the results. The principal investigator of WAPPS-Hemo, Dr Alfonso Iorio, agrees to share ownership of the database and authorship on any publication stemming from the project. Clinicians contribute to the repository by submitting to the website as few as 3 to 4 factor levels per patient along with demographic information. The appropriate PopPK control file is selected for the brand of factor concentrate identified in the patient data file. The online PopPK engine automatically estimates the relevant individual PK parameters. Following expert validation, a patient report is generated and sent to the clinician that includes the time when the factor level reaches a specified value, for example 0.05, 0.02 or 0.01 international units (IU) per mL, along with credibility intervals.

### Objectives

The primary objective of this report is to outline the methods for developing PopPK models on dense FVIII and FIX data obtained from the Data Sources to better understand the relationship between blood plasma concentration and time for each molecule investigated. PopPK model estimates will be entered as priors in subsequent Bayesian post hoc analyses to predict the most reliable function between blood plasma concentration and time for patients with sparse data. This function will be used to inform clinicians when the next dose of a particular FVIII or FIX molecule should be administered.

## Methods

### Data Sources

Dense individual PK data on 878 participants using 21 different molecules from 17 different sources have been collected as part of industry-sponsored or investigator-driven studies. Most of the data in the derivation cohort are provided as both clotting and chromogenic assay results, but we plan to model exclusively with data from clotting assays, as the data received from participating clinical sites is almost uniquely of this type. Characteristics of some of the dense data that has been obtained for the WAPPS-Hemo project are summarized in Table 1. All datasets reported the age and weight of the participants; certain studies also reported additional covariates such as hematocrit, von Willebrand factor levels, and blood type, which can be tested during covariate analysis. In addition, individual patient data is being collected continuously through the WAPPS-Hemo co-investigator network that currently has 47 active hemophilia treatment centers registered. To date, close to 300 profiles have been submitted.

**Table 1:** Summary of some dense data used for initial population modeling.

Brand	Type	N^a^	Age, years	Weight, kg	Hematocrit included	vWF^b^ level included	Blood type included
Advate	FVIII^c^ recombinant	25	15-62	53.8-127.4	No	Yes	No
Alprolix	FIX^d^ recombinant	129	12.1-71.5	45.0-186.7	Yes	No	No
Benefix	FIX recombinant	80	4.3-58.5	17.9-186.7	Yes	No	No
Eloctate	FVIII recombinant	167	12-65	42.0-129.2	No	Yes	Yes
Kogenate	FVIII recombinant	40	13.0-56.1	47.4-124.2	No	No	No
Kovaltry	FVIII recombinant	23	12-51	46.3-124.2	No	No	No
Xyntha	FVIII recombinant	30	14-57	50.7-117.2	No	No	No

^a^N: number of participants.

^b^vWF: von Willebrand factor.

^c^FVIII: clotting factor FVIII.

^d^FIX: clotting factor FIX.

### Dataset Assembly

#### Rich Dataset

Rich data used for PopPK modeling are provided by the data sources. These data will be received in various software packages and in a variety of formats, so they will be re-formatted into a standard comma-separated values (CSV) file for input into the PopPK modeling software, NONMEM (v 7.3.0; ICON Development Systems, Ellicott City, MD, US). Where possible, the dataset will consist of the variables shown in Table 2. AMT, DV, TIMEH, AGE, and BW are required to be provided by the data source. The optional covariates (HT, VWF, RACE, BTYPE, and HCT) are collected if possible.

#### Structure of the NONMEM Dataset

The record for each patient is organized as follows. The first record is used to read in the pre-dose amount, which accounts for the patient’s endogenous factor level and any residual factor from a previous dose, if measured. The TIMEH entry for the first record is set to zero, and this is the reference point for the time for all subsequent records in the dataset. The first record also contains the BASELINE value, which corresponds to the patient’s endogenous factor level. If a baseline level of the factor was measured, the measured value is entered; if not, a baseline value of 0.005 IU/mL (0.5% of normal factor activity) is assumed.

The second record is used to read in the dose administered (AMT). For this entry, the TIMEH column contains the time (in hours) that was required to administer the dose (eg, 0.1666 for a 10-minute administration). The amount and time are used to calculate the rate (RATE=AMT/TIMEH). For all subsequent records, AMT and RATE are set equal to zero.

The third record contains the first valid observation of the plasma concentration and subsequent records contain subsequent valid observations of the plasma concentration. The one exception to this is records following a valid observation that refer to samples that are below the limit of quantification (BLQ). Because the information from these different events (eg, PREDOSE, BASELINE, concentration observations, and BLQ events) needs to be handled in different ways, indicator variables MDV3 and MDV5 are included to designate how each entry should be used.

**Table 2:** Typical variables in NONMEM datasets.

Variable		Description	Units
**Required variables**			
	CID	Patient identification number	Positive integer
	OCC	Dose occasion	Positive integer
	TIMEH	Time for each concentration measurement from start of bolus	Hours or fraction of hours (minimum of 4 decimal places)
	AMT	Total dose	IU^a^
	RATE	Rate of entry of drug: AMT/TIMEH	IU/h
	DV	Plasma concentration of valid observation or BLQ^b^	IU/L
	AGE	Age	Positive integer, years
	BW	Weight	Positive integer, kilograms
	EVID	Event identification variable	Positive integer (0=valid observation, 1= dose, 3=BLQ observation)
	DOSE	AMT/BW	Positive number, IU/kg
	PREDOSE	Plasma concentration at time of start of bolus	Zero or positive integer if measured, –1 if not measured (IU/L)
	MDV5	Missing dependent variable	0=valid observation; 1=dose or BLQ observation; MDV5=MDV when no BLQ
	BASELINE	Endogenous plasma concentration	Positive integer if known, –1 if not known, IU/L
	BLQ	Below limit of quantification	≤ 0=non BLQ measurement, positive integer=BLQ value, IU/L
	MDV3	Missing dependent variable	0=valid observation or BLQ; 1=dose; MDV3=MDV when BLQ is present
**Optional covariates**			
	HT	Height	Positive integer, centimeter
	VWF	von Willebrand factor	Percentage
	RACE	Race	Positive integer (1=White, 2=Black, 3=…)
	BTYPE	Blood type	Positive integer (1=A, 2=B, 3=AB, 4=O)
	HCT	Hematocrit	Percentage

^a^IU: international unit.

^b^BLQ: below the limit of quantification.

### Data Checking

#### Errors and Missing Data

Prior to analyzing the data, the integrity of the data will be scrutinized to identify potential data errors. Errors can exist for a number of reasons. For example, following a dose, plasma concentrations typically decline with time so if a plasma concentration for a record is higher than the plasma concentration for a previous record, that record will be flagged to be checked. If any data are missing they will be flagged to be checked. Similarly, outlying covariate values for continuous variables (eg, AGE, BW, or HT) will be flagged to be checked. Any categorical variable that has a value that is not expected will be flagged to be checked. Duplicate records within a patient’s data will also be flagged.

#### Procedures for Handling Data Errors

All potentially erroneous data will be reported and discussed. If a resolution to the error is forthcoming, it will be documented and the appropriate changes will be made to the dataset. If no resolution is found, the error will be documented and the data will be excluded from subsequent analyses.

### Data Modeling Methods

#### Software, Subroutines, and the Handling of Data Below the Limit of Quantification

Nonlinear mixed effects modeling and Bayesian post hoc estimations will be completed in NONMEM and PDx-Pop (v 5.10; ICON Development Systems, Ellicott City, MD, US). PopPK modeling will be performed using the first order conditional estimation with interaction (FOCEI) method. The ADVAN and TRANS subroutines for each model, which specify the model structure and parameterization, respectively, are shown in Table 3.

**Table 3:** NONMEM subroutines used to implement kinetic equations for linear models following intravenous administration.

Model	ADVAN subroutine	TRANS subroutine
1-compartment	ADVAN1	TRANS2: CL, V
2-compartment	ADVAN3	TRANS4: CL, V1, Q, V2
3-compartment	ADVAN11	TRANS4: CL, V1, Q2, V2, Q3, V3

Severe hemophilia patients have, by definition, an endogenous coagulation factor level below 0.01 IU/mL, which is also often cited as the limit of quantification (LOQ) for coagulation activity assays [27-29]. As a result, trough concentrations are often BLQ and several methods exist for the handling of samples that are BLQ. Simpler methods exclude BLQ data or replace these points with LOQ/2; this protocol makes use of the M3 method described by Beal in 2001 [30].

#### Structural Model Building

The first step in model development will be a naïve pooled analysis, which allows for preliminary exploration of model structure and mean estimates of PK parameters. Further definition of the model structure (ie, number of compartments) will be determined using a combination of graphical techniques and numerical goodness-of-fit measures. Models will be evaluated using an objective function value based on a summation of the residual error. One model is considered to be superior to a similar hierarchically well-formulated model with one more degrees of freedom if the objective function decreases by 3.84 units or more, based on the assumption of a chi squared (χ^2^) distribution. Models will also be evaluated using diagnostic plots (Textbox 2).

**Diagnostic plots used to evaluate the models.**

- Observed values vs individual/population predicted values
- Conditional weight residuals (CWRES) vs predicted values
- CWRES vs time
- Observed and predicted values vs time
- Normal QQ-plots
- CWRES histogram
- Eta histograms
- Population covariate plots

In the event that it is difficult to determine which structure best characterizes the data, it may be helpful to fit each subject individually to explore the reasons for unexplained variability. For example, some factor concentrates may exhibit different structures between patients, which may in turn require estimates to be derived from both models followed by a comparison of the effects on population estimates and individual dosing decisions; as a rule of thumb, we will always take the most conservative approach.

**Figure 2:**
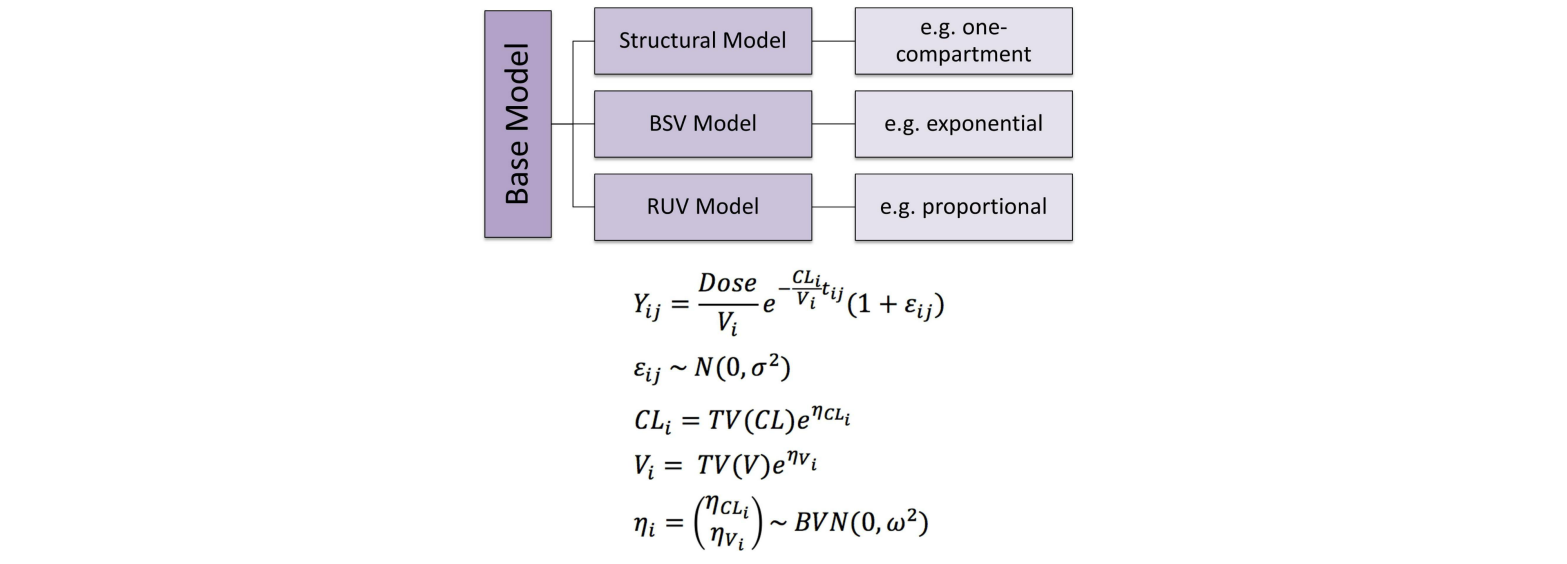
Illustration of the various components of the base model for a one-compartment model with exponential between subject variability and proportional residual unexplained variability.

**Figure 3:**
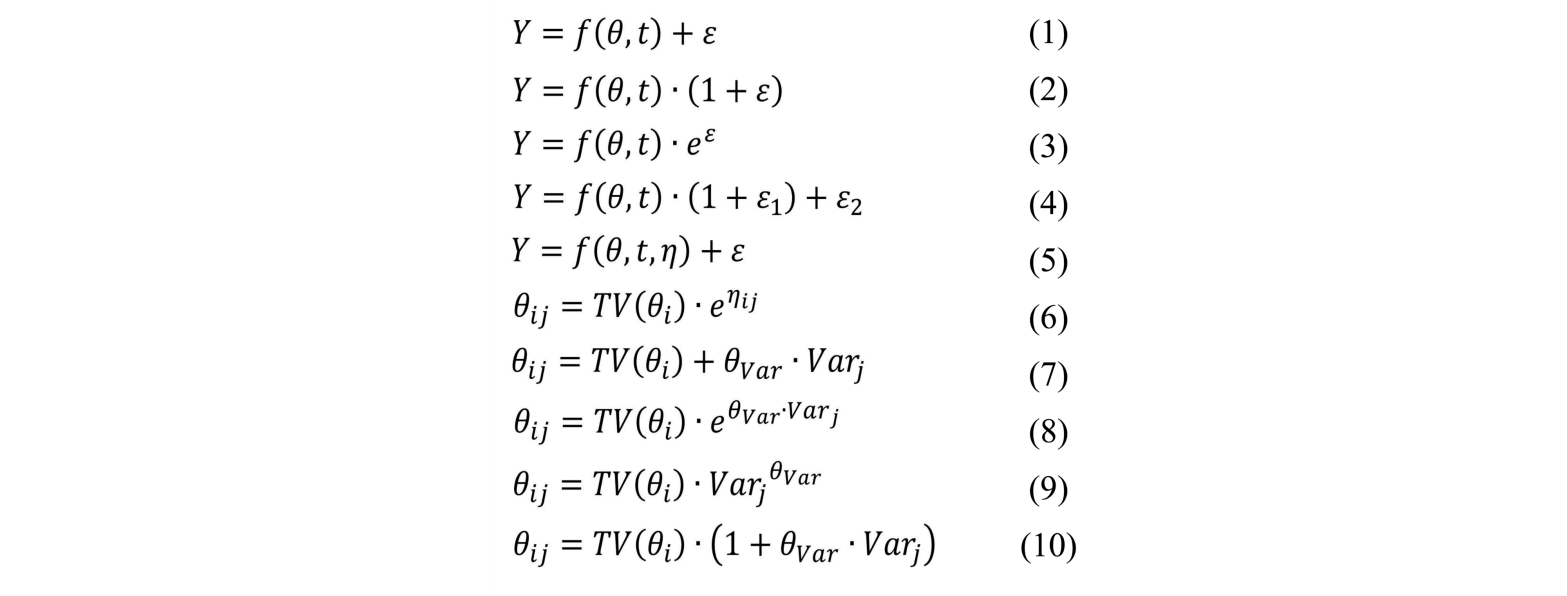
Equations for defining different aspects of the population pharmacokinetic models including residual unexplained error (1-4), between subject variability (5-6), and covariates (7-10).

The goal of PopPK is to describe the concentration-time profile for each subject using a series of mathematical equations in a hierarchical manner (Figure 2). Observed concentrations are expressed as a function of an individual’s PK parameters (*θ*) and time (*t*), with a residual error term (ε) to account for unexplained variability within the individual (Figure 3, Equation 1).

The appropriate structure for the residual unexplained variability (RUV, ε) will be determined using graphical goodness-of-fit plots (including histograms of the residuals, normal QQ plots, and plots of the residuals vs predicted values) and numerical measures (such as objective function value and shrinkage). Possible models for the RUV are shown in Figure 3 (Equations 1-4) where ε’s are independent and normally distributed, with a mean of zero and variance of σ^2^. The combined additive-proportional error model (Figure 3, Equation 4) is most commonly used in PK modeling.

From a population of participants, an estimate of the typical value of the relevant PK parameters can be obtained. A new parameter, η, can then be used to describe how an individual’s parameter deviates from the typical value (ie, the BSV, Figure 3, Equation 5).

The BSV (*η*) of PK parameters will be estimated using the relationship shown in Equation 8 (Figure 3) where *θ*_ij_ is PK parameter *i* for the *j*^th^individual, *TV(θ*_i_) is the population mean value of the parameter, *η*_ij_ is the subject-specific deviation from the population mean of PK parameter *i* for individual *j*. The *η* ’s are normally distributed, with a mean of zero and a variance-covariance matrix, ω^2^. The functional form chosen for the BSV is based on the assumption that PK parameters are log-normally distributed. In the event that this model does not provide a good fit, other functional forms may be explored.

Initially, BSV will be included on all PK parameters, and the necessity of all these terms will be investigated both graphically and using formal hypothesis tests. Once the significant random effects have been identified, the structure of the variance-covariance matrix (ω^2^) can be explored. All prior model development assumes a diagonal variance-covariance matrix (ie, no correlation between random effects). Comparing models with diagonal and unstructured variance-covariance matrices will test for the correlation between random effects.

#### Covariate Model Building

In order to minimize the unexplained portion of the BSV, covariates will be added to the model. Potential covariate relationships will first be explored by examining plots of the included *η* ’s against each covariate. From these plots, the covariates that are most likely to be significant can be identified, and then tested formally in the model. Various functional forms describing the relationship between the covariates and the PK parameters are possible. Examples of commonly used functional forms are shown in equations 7-10 of Figure 3 where *Var*_j_ is the covariate value for individual *j* and *θ*_Var_ describes the magnitude and direction of the correlation between the covariate and parameter. Often, covariates such as weight and age will be centered or scaled by the mean or median; this assists in the interpretation of the estimates and helps to stabilize the estimation procedure. Covariates will be added to the model in a stepwise manner by considering formal hypothesis test results, precision of the parameter estimates and graphical techniques.

All model-building datasets include age and weight as parameters, but data for certain brands of factor concentrate could also include height, hematocrit, von Willebrand factor level and blood group as possible covariates. Where available, these covariates can be tested in model development and, if fitting the above criteria for retention, be included in the final model. However, the choice of model would need to take into account the fact that clinicians using WAPPS-Hemo for individual PK estimations are required to include age and weight when requesting PK estimates from the WAPPS-Hemo platform, whereas inclusion of other covariates listed above is optional. Therefore, it is possible that a covariate may significantly influence the PK of a molecule, but may not be recorded at the clinical site. In order to reconcile significant covariates and available information, multiple models may be produced for a single molecule and a process algorithm for determining which model to use in a given situation will be incorporated into the WAPPS-Hemo platform. The decision tree may also incorporate different structural models for molecules that behave differently between subjects, and the model that provides the most precise estimates will be selected. In all cases, the clinician will receive a single report corresponding to whichever model was chosen for the data provided.

### Population PK Model Evaluation

The first step in model evaluation includes the use of the diagnostic plots outlined above to ensure that all model assumptions are being met (eg, independent and normally distributed residual error, normally distributed random effects). Also, metrics such as the condition number and the variance inflation factor may be used to assess collinearity. A bootstrap analysis will also be performed to ensure that the model is stable and provides precise estimates for all parameters.

Next, the models will be evaluated using cross-validation techniques with the rich data. Either the holdout method or a *k*-fold cross-validation technique will be employed, depending on the size of the dataset in question. The bias and accuracy of the models will be assessed using the metrics of mean error and mean squared error.

Following evaluation with rich data, the models will be validated using sparsely sampled data to ensure that they perform adequately with the type of data that will be provided by clinicians using the WAPPS-Hemo platform. Validation with sparse data presents some challenges, since the typical methods discussed above cannot be employed. However, a number of strategies for evaluating models using sparse data have been reported. These include using a subset of a complete sampling scheme to compare performance [31,32], performing Monte Carlo simulations [32], and examining predictive distributions of the observed concentrations [33]. Our preferred method is the use of a subset of a complete set of samples.

### Bayesian Post Hoc Model

**Figure 4:**
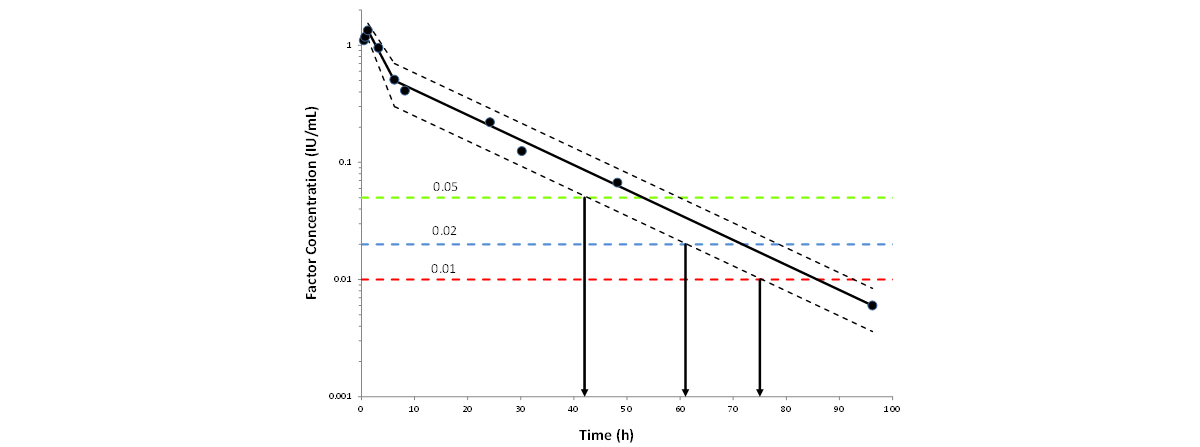
Factor concentration as a function of time (symbols: patient data, black line: predicted individual pharmacokinetic profile) where time to the lower 95% credibility interval bound for each of the 0.05 (green line), 0.02 (blue line) or 0.01 (red line) IU/mL thresholds is reported to the clinician. Time 0 represents time of dose initiation.

Bayesian estimations will also be performed in NONMEM, using the parameter estimates from the PopPK models as informative priors for the relevant PK parameters (eg, volume of distribution, clearance). This step will use the same model structures and estimation methods as previously described, and will handle the presence or absence of PREDOSE, BASELINE, and BLQ values in the same manner as outlined above. From the output files, the time from dose initiation to various concentrations (eg, 0.05, 0.02 or 0.01 IU/mL) or the concentration at different times (eg, 24, 48, and 72 h) can be reported with the accompanying 95% credibility intervals. The times reported to the clinician will be the times at which the lower boundary of the 95% credibility interval for concentration first reaches each of these three concentration thresholds (Figure 4). For concentrations at specified times, the credibility interval would report the lower and higher concentration value estimated at that given time.

We have opted to use the credibility interval as the most efficient and understandable way to report the amount of “shrinkage” of the patient data to the population model. The interval will be larger or smaller depending upon the amount of information that is used either from the population (ie, larger band where most values within the population are possible for the patient) or the individual (ie, smaller band where more rich patient data reduces variability). The Bayesian approach used allows this variability to vary across different segments of the curve, being large where no or little information is provided and small where informative points are provided.

### Reporting

A comprehensive PopPK report will be assembled for each brand-specific model that is developed, according to the Food and Drug Administration (FDA) guidance on PopPK reporting [34]. The recommended sections included are shown in Textbox 3. These guidelines apply to reports submitted to the FDA, which are not directly available to the public; however, a close approximation of the reports we will be generating for each model for peer-review publication is provided by Rajagopalan and Gastonguay [35].

**Recommended sections included in the comprehensive population pharmacokinetics report.**

- Summary
- Introduction
- Objectives, Hypotheses and Assumptions
- Materials and Methods
  - Assay
  - Data
  - Data Analysis Methods
- Results
- Discussion
- Application of Results
- Appendix

## Results

Dense PK data has been collected for more than 20 brands of factor concentrate. Models have been developed for all but three molecules, and we expect to receive data for one additional molecule in early 2017. All models will undergo further refinement and validation, and be submitted for publication in 2017. From the WAPPS-Hemo co-investigator network, we have collected 300 PK assessments to date and expect to reach the 500-assessment mark by early 2017.

## Discussion

### Risks and Barriers

The main risk associated with the use of the WAPPS-Hemo service is the possibility that the specific patient is outside of the covariate space used to build the models. In such cases, the individual estimated PK parameters may be imprecise or essentially “wrong” and could result in suboptimal treatment decisions. In light of this, we plan to implement risk minimization procedures. First, we provide both average estimates as well as their associated credibility intervals. In cases where the patient is outside of the model development space, we expect the intervals to be large such that clinical usage of the predictions is discouraged. Second, each forecasted PK is reviewed individually by an expert and appropriate warnings will be added as needed. Third, as a general policy for WAPPS-Hemo users, we recommend that the PK prediction is used as a tool to speed up treatment optimization. To this end, we recommend prospective testing with sampling around specific times that would be valuable in decreasing uncertainty.

One of the main goals of the WAPPS-Hemo program is to eliminate barriers to the uptake of an individualized PopPK-driven approach to hemophilia treatment. By adopting this tool, clinicians require fewer blood samples and circumvent the complex calculations usually needed to implement a tailored dosing strategy. However, the current output report may be a potential hindrance. Although the report contains times to critical factor levels as well as concentrations at convenient time points, these results only pertain to the dose that was administered. A proposed clinical module will allow clinicians to input two parameters among dose, frequency, and desired factor level to calculate the third. This additional functionality will allow those that treat hemophilia to evaluate the theoretical effect of changing dose and frequency on future plasma levels in real time without having to submit multiple profiles through the WAPPS-Hemo platform.

### Conclusions

In summary, the WAPPS-Hemo service is predicated on valid PopPK models. This report focuses on describing the process for model development and evaluation, which all brand-specific models will undergo. Rich data has been, and continues to be, the main source of data for model development. However, as clinical sites contribute sparse data to the repository, a greater breadth of PK data and covariates will allow for continuous quality improvements in the models.

## Abbreviations

BLQ: below the limit of quantification
BSV: between subject variability
CWRES: conditional weight residuals
FIX: clotting factor IX
FVIII: clotting factor FVIII
LOQ: limit of quantification
PK: pharmacokinetic
PopPK: population pharmacokinetics
WAPPS-Hemo: Web-Accessible Population Pharmacokinetic Service - Hemophilia
WSV: within subject variability
